# Assessment of a risk warning model for depression in lymphedema patients after breast cancer surgery

**DOI:** 10.3389/fmed.2026.1749589

**Published:** 2026-05-08

**Authors:** Linli He, Yanyan Feng, Hui Li, Youcheng Huang, Yonghong Lu, Hong Wang, Congying Zhang, Xi Yang

**Affiliations:** 1 Department of Dermatology, West China School of Medicine, Chengdu Second People’s Hospital, Sichuan University Affiliated Chengdu Second People’s Hospital, Sichuan University, Chengdu, Sichuan, China; 2 Department of General Surgery, West China School of Medicine, Chengdu Second People’s Hospital, Sichuan University Affiliated Chengdu Second People’s Hospital, Sichuan University, Chengdu, Sichuan, China; 3 Department of Cardio-Thoracic Surgery, West China School of Medicine, Chengdu Second People’s Hospital, Sichuan University Affiliated Chengdu Second People’s Hospital, Sichuan University, Chengdu, Sichuan, China

**Keywords:** breast cancer, depression, factor, lymphedema, power analysis

## Abstract

**Objective:**

To identify independent risk factors for depression in breast cancer patients with postoperative lymphedema and develop an early warning model for depression prevention.

**Methods:**

120 breast cancer patients with postoperative lymphedema were divided into depression (*n* = 52) and non-depression (*n* = 68) groups based on the Self-Rating Depression Scale (SDS) administered 3 months after lymphedema onset. Data on demographics, clinical parameters, and social and cognitive functions were collected. Logistic regression analysis was used to identify independent risk factors. An early warning model was constructed and validated using temporal consecutive split-sample validation, with the first 120 consecutively enrolled patients constituting the development cohort and the subsequent 60 consecutive patients constituting the internal validation cohort.

**Results:**

Depression incidence was 43.33%. Univariate analysis showed significant differences in living arrangements, family income, surgical side, lymphedema severity, body image, pain level, sleep disturbances, lymphedema knowledge, and social support. Multivariate analysis identified low family income (<3000 yuan), poor body image, and low social support as independent risk factors. External validation in the temporal consecutive internal validation cohort (*n* = 60) confirmed these findings (AUC: 0.629, 95% CI: 0.487–0.772, sensitivity: 82.61%, specificity: 43.24%).

**Conclusion:**

Breast cancer patients with postoperative lymphedema demonstrate high rates of depression, particularly among those with low family income, poor body image, and low social support. These factors represent potential targets for screening and intervention; however, causal relationships require confirmation through longitudinal and interventional studies.

## 1 Introduction

Breast cancer, as a common malignant tumor that plagues women worldwide, has a high incidence rate. According to data from the International Agency for Research on Cancer in 2019, breast cancer ranks first among cancers in women globally, accounting for 24.2%, of which 52.9% are from developing countries (1). With the development of medical technology, surgical treatment is now the main approach, which is beneficial for extending the survival period of patients. Relevant data indicates that the 5-years survival rate for early-stage breast cancer patients after surgery can reach 76%–92% (2). However, patients are more likely to develop lymphedema after surgery. A 2-years prospective follow-up study has confirmed that the incidence of lymphedema after breast cancer surgery increases over time, rising from 4.4% 1 month after surgery to 32.5% 2 years after surgery (3). As a serious complication after breast cancer surgery, lymphedema, although it does not affect the patient’s life expectancy, can severely affect the patient’s limb shape and function, reduce the quality of life, and lead to anxiety and depression, making it difficult for patients to reintegrate into society quickly (4). However, up to now, there has been no systematic research on the factors influencing depression in breast cancer patients with postoperative lymphedema. Recently, predictive modeling approaches have demonstrated feasibility for identifying cancer-related depression risk in various oncological populations (5). However, these models have not been specifically validated in breast cancer patients with postoperative lymphedema, a distinct clinical population with unique physical and psychological stressors. The development of targeted risk assessment tools for this specific complication remains essential for early identification and intervention. This paper collects clinical data to conduct univariate and multivariate analyses using the Logistic regression model in order to identify the independent risk factors for depression in breast cancer patients with postoperative lymphedema. The aim is to construct a depression risk screening model for breast cancer patients with postoperative lymphedema, so as to identify high-risk patients who may benefit from early psychological assessment and support.

## 2 Materials and methods

### 2.1 General information

This study employed temporal consecutive split-sample validation for model development and validation. Consecutive eligible patients were enrolled from February 2023 to August 2024. The first 120 patients constituted the model development cohort, and the subsequent 60 patients constituted the internal validation cohort. This sequential allocation ensured the validation data were not used in model development while maintaining identical enrollment criteria and assessment protocols across both cohorts. The inclusion criteria were as follows: ① meeting the relevant criteria in the “Guidelines and Standards for the Diagnosis and Treatment of Upper Limb Lymphedema after Breast Cancer Surgery” (6); ② satisfying the indications for breast-conserving surgery, modified radical mastectomy, and axillary lymph node dissection; ③ obtaining informed consent from the patient and their family members. Patients were enrolled upon lymphedema diagnosis and underwent close follow-up. All clinical and demographic variables were recorded at baseline (lymphedema diagnosis). Depression status was assessed using the SDS at the 3-months follow-up visit post-lymphedema onset, a timepoint selected to capture the critical adaptation period when patients transition from acute postoperative care to chronic lymphedema management.

The exclusion criteria were: ① having other malignant tumors; ② having local recurrence or distant metastasis; ③ having cognitive disorders. The study was approved by the ethics committee.

Sample size justification: the sample size was determined based on the minimum events per variable (EPV) principle for multivariable logistic regression analysis. With 9 candidate predictor variables and an anticipated depression prevalence of 40% based on prior literature (7, 8), we aimed for a minimum EPV of 5–10 to ensure stable coefficient estimation (9). This required a minimum of 45–90 depression events, translating to a total sample size of 113–225 patients. We conservatively targeted 120 patients for the development cohort to account for potential missing data and to enable temporal split-sample validation with a 2:1 ratio (development: validation).

*Post hoc* power analysis: based on the final multivariable model with three significant predictors (family income, body image, and social support), a *post hoc* power analysis was conducted using G*Power 3.1.9.7. With an observed effect size (odds ratio) of 2.585 for the smallest significant predictor (social support), an alpha level of 0.05, and the achieved sample size of 120 in the development cohort (52 depression events), the study achieved 82.3% power to detect this effect. For the largest predictor (body image, OR = 3.869), power exceeded 95%. These calculations confirm adequate statistical power for detecting clinically meaningful associations.

Rationale for 3-months assessment window: the 3-months post-lymphedema onset timeframe was selected for several methodological and clinical reasons. First, this period represents the critical adaptation phase when patients transition from acute postoperative recovery to chronic lymphedema management, a transition associated with heightened psychological vulnerability (4, 10). Second, a 3-months minimum duration ensures that lymphedema is established as a chronic condition rather than transient postoperative edema, which typically resolves within 6–8 weeks (6). Third, this timeframe minimizes confounding from acute surgical recovery and adjuvant therapy (chemotherapy/radiotherapy), as the majority of patients complete primary cancer treatment within 2–3 months post-surgery. Fourth, assessing depression at a consistent 3-months post-lymphedema diagnosis point standardizes the exposure duration across patients, reducing temporal variability in the predictor-outcome relationship.

Exclusion of acute treatment effects: to further ensure that depression outcomes were attributable to lymphedema rather than acute cancer treatment effects, we excluded patients who were actively receiving chemotherapy or radiotherapy at the time of lymphedema diagnosis (*n* = 8 excluded). All included patients had completed primary cancer treatment (surgery ± adjuvant therapy) at least 4 weeks prior to lymphedema onset. This washout period allows for dissipation of acute treatment-related mood disturbances while capturing the specific psychological burden of chronic lymphedema.

### 2.2 Grouping criteria

At the 3-months follow-up assessment, patients were grouped according to Self-Rating Depression Scale (SDS) scores (11). The SDS consists of 20 items, each rated on a 4-point Likert scale from 1 to 4. The raw score is calculated by multiplying the total score by 1.25, with a maximum score of 100. The cutoff value is 53. Patients with a score of 53 or higher were considered to have depression and were assigned to the depression group (*n* = 52), while those with a score below 53 were assigned to the non-depression group (*n* = 68).

### 2.3 Observation indicators

Demographic information, clinical parameters, and indicators related to social and cognitive functions were collected. Specifically:

1. Demographic Information:
  a. Age (≥60 years, <60 years)
  b. Living Arrangements (Living alone, Living with others)
  c. Education Level (Junior high school and below, Senior high school and above)
  d. Marital Status (Married, Unmarried, Divorced or Widowed)
  e. Family Monthly Income (≥3000 yuan, <3000 yuan)
2. Clinical Parameters:
  a. Comorbidities (Yes/No)
  b. Surgical Method (Breast-conserving surgery, Modified radical mastectomy)
  c. Surgical Side (Left, Right)
  d. Axillary Lymph Node Dissection (Yes/No)

Severity of Lymphedema [Based on reference (12)]: assessment of the patient’s lymphedema status was conducted by a certified Lymphedema Therapist using the standardized upper limb circumference measurement method (in accordance with the *Chinese Nursing Association Group Standard 2021-05-01*). Measurement sites and procedure: Bilateral limb circumferences were measured on-site using a non-elastic tape measure at the following locations: the first web space (between the thumb and index finger), wrist crease, 10 cm below the elbow crease (antecubital fossa), elbow crease, 10 cm above the elbow crease, and axillary level; measurements were taken every 10 cm, with each site measured twice and the average value recorded. Severity assessment: “Mild edema: affected side minus unaffected side = 2–4 cm; Moderate edema: affected side minus unaffected side = 4–6 cm; Severe edema: affected side minus unaffected side > 6 cm.”

1. Indicators Related to Social and Cognitive Functions:

The Modified Body Image Scale (MBIS) includes 8 assessment items covering femininity, appearance-related insecurity (lack of confidence), body satisfaction, etc. Each item uses a 3-point Likert scale (scoring 1–3), with a total possible score of 24. Using the median score of 12 as the cutoff, scores of 12–24 are classified as low self-image, while scores of 0–12 are classified as high self-image. Based on reference (13), assessment was conducted using the Modified Body Image Scale (MBIS); using the median score of 12 as the cutoff, “scores of 12–24 were considered as low self-image, and 0–12 as high self-image.”

1.
  a. Sexual Dysfunction [Based on reference (14), assessed using the Female Sexual Function Index (FSFI), with a score of ≥25 indicating normal function and a score below 25 indicating dysfunction]
  b. Pain Level [Based on reference (15), assessed using the Visual Analog Scale (VAS), with 0–3 indicating no to mild pain and 4–10 indicating moderate to severe pain]
  c. Sleep Disturbances (Yes/No)
  d. The lymphedema knowledge score is supplemented as follows: “Based on reference (16), assessment was conducted using the Breast Cancer Lymphedema Related Knowledge Questionnaire, comprising 20 true/false questions; correct responses were scored 1 point and incorrect responses 0 points, with scores ≥ 12 considered as good mastery and scores < 12 considered as poor mastery.”
  e. Social Support “Based on reference (17), assessment was conducted using the Social Support Rating Scale (SSRS), with a total score of 40; scores ≥ 20 was considered as high social support, while scores < 20 were considered as low social support.” It comprises 10 items across three dimensions: objective support (4 items), subjective support (3 items), and social support utilization (3 items), with a total score of 40. Scores ≥ 20 are considered high social support, while scores < 20 are considered low social support.

### 2.4 Statistical analysis

Data were analyzed using SPSS version 22.0 (IBM Corp., Armonk, NY). Categorical variables were expressed as frequencies (percentages) and compared between groups using Chi-square tests or Fisher’s exact tests (when expected cell frequency < 5). Continuous variables were expressed as median (interquartile range) due to non-normal distribution and compared using Mann-Whitney U tests. All demographic, clinical, and psychosocial variables were first examined using univariate logistic regression. Variables with *P* < 0.05 were considered eligible for multivariate analysis. Significant univariate predictors were entered simultaneously into a multivariate logistic regression model using the Enter method. A depression risk early warning model was then constructed for these patients. The diagnostic efficacy of the model was assessed using the receiver operating characteristic (ROC) curve. Model calibration was evaluated to assess the agreement between predicted probabilities and observed outcomes. Calibration was quantified using the calibration intercept (ideal = 0) and calibration slope (ideal = 1) from a logistic regression of observed outcomes on predicted log-odds. A calibration intercept of 0 indicates no systematic over- or under-prediction; a calibration slope of 1 indicates appropriate spread of predictions. The Brier score was calculated as the mean squared difference between predicted probabilities and observed binary outcomes (range 0–1, where 0 indicates perfect prediction). Calibration plots displaying predicted probabilities against observed frequencies (grouped by deciles of predicted risk) were generated for visual assessment. These metrics were calculated for both the development cohort and the internal validation cohort to evaluate calibration performance and potential overfitting. A *p*-value of less than 0.05 was considered to indicate statistical significance.

Missing data handling: complete case analysis was used; patients with missing data for any predictor or outcome variable were excluded from analysis.

Variable selection strategy: all variables with *P* < 0.05 in univariate analysis were simultaneously entered into the multivariate model using the Enter method. No variable selection algorithms (e.g., stepwise) were employed to avoid data-driven overfitting. Variance inflation factors (VIF) were calculated to assess multicollinearity; all VIF values were <2.0, indicating no significant multicollinearity.

Overfitting assessment: given the limited number of outcome events (*n* = 52) and 9 candidate predictors (EPV ≈ 5.8), we employed multiple strategies to mitigate overfitting: (1) temporal consecutive split-sample validation with separate internal validation cohort; (2) bootstrapping (500 resamples) to estimate optimism-corrected performance; (3) reporting calibration metrics to assess reliability of predicted probabilities. The similarity in sensitivity between development (86.54%) and validation (82.61%) cohorts suggests minimal overfitting.

## 3 Results

### 3.1 Incidence of depression in breast cancer patients with postoperative lymphedema

Among the 120 breast cancer patients with postoperative lymphedema, 52 patients had depression, with an incidence rate of 43.33%.

### 3.2 Univariate analysis of demographic information, clinical parameters, and indicators related to social and cognitive functions between the two groups

The results of the univariate analysis showed that, except for age, education level, marital status, comorbidities, surgical method, axillary lymph node dissection, and sexual dysfunction, there were statistically significant differences between the two groups in terms of living arrangements, family monthly income, surgical side, severity of lymphedema, body image, pain level, sleep disturbances, knowledge of lymphedema, and social support (*P* < 0.05) (Table 1).

**TABLE 1:** Univariate analysis of demographic information, clinical parameters, and social and cognitive function-related indicators between the two groups [*n* (%)].

Factors		Depression group (*n* = 52)	Non-depression group (*n* = 68)	χ^2^	*P*
Age (No)	≥60 Yr	18	20	0.369	0.544
	<60 Yr	34	48		
Living arrangement (No)	Living alone	15	9	4.488	0.034
	Living together	37	59		
Educational level (No)	Junior high school and below	21	23	0.546	0.460
	High school and above	31	45		
Marital status (No)	Married	41	54	0.062	0.951
	Unmarried	3	4		
	Divorced or widowed	8	10		
Family monthly income (No)	≥3000 Yuan	12	32	7.298	0.007
	<3000 Yuan	40	36		
Comorbidities (No)	Y	9	6	1.939	0.164
	N	43	62		
Surgical procedure (No)	Partial mastectomy	38	45	0.658	0.417
	Modified radical mastectomy	14	23		
Surgical side (No)	Dominant side	36	29	8.388	0.004
	Non-dominant side	16	39		
Axillary lymph node dissection (No)	Y	32	46	0.483	0.487
	N	20	22		
Severity of lymphedema (No)	Mild edema	14	40	12.116	0.001
	Severe edema	38	28		
Body image (No)	High	7	31	14.055	0.000
	Low	45	37		
Sexual dysfunction (No)	Normal	19	33	1.725	0.189
	Impairment	33	35		
Pain level (No)	None to mild	13	38	11.500	0.001
	Moderate to severe	39	30		
Sleep disturbance (No)	Y	31	28	4.009	0.045
	N	21	40		
Knowledge of lymphedema (No)	Good	10	31	9.101	0.003
	Poor	42	37		
Social support (No)	High	16	42	11.336	0.001
	Low	36	26		

Y, Yes; N, No; Yr, Year.

### 3.3 Multivariate analysis of logistic regression model for depression in breast cancer patients with postoperative lymphedema

The results of the multivariate analysis using the Logistic regression model showed that the independent risk factors for depression in breast cancer patients with postoperative lymphedema were family monthly income (<3000 yuan) (OR: 2.823; 95% CI: 1.065∼7.482), poor body image (OR: 3.869; 95% CI: 1.195∼12.527), and low social support (OR: 2.585; 95% CI: 1.004∼6.653) (Table 2).

**TABLE 2:** Multivariate analysis of logistic regression model for predicting depression in patients with lymphedema after breast cancer surgery.

Factors	B	SE	Waldχ^2^	*P*	OR (95% CI)
Constant	−3.843	0.772	24.795	0.021	–
Living arrangement (living alone)	0.407	0.619	0.433	0.511	1.503 (0.446∼5.060)
Family monthly income (<3000 Yuan)	1.038	0.497	4.354	0.037	2.823 (1.065∼7.482)
Surgical side (dominant side)	0.514	0.468	1.206	0.272	1.671 (0.668∼4.180)
Severity of lymphedema (severe)	0.561	0.500	1.256	0.262	1.752 (0.657∼4.673)
Body image (low)	1.353	0.599	5.096	0.024	3.869 (1.195∼12.527)
Pain level (moderate to severe)	0.276	0.505	0.299	0.585	1.318 (0.490∼3.546)
Sleep disturbance (present)	0.363	0.479	0.574	0.449	1.438 (0.562∼3.676)
Knowledge of lymphedema (poor)	0.434	0.538	0.652	0.419	1.544 (0.538∼4.432)
Social support (low)	0.950	0.482	3.877	0.049	2.585 (1.004∼6.653)

### 3.4 Construction of the risk early warning model and ROC curve analysis for predicting depression in breast cancer patients with postoperative lymphedema

Based on the multivariate logistic regression, the final predictive model included three independent risk factors. The model equation is:

Logit(P) = −3.843 + (1.038 × Family Income) + (1.353 × Body Image) + (0.950 × Social Support)

Where:

1. Logit(P) = ln[P/(1−P)], the natural log of the odds of depression
2. Intercept (β_0_) = −3.843
3. Family Income coefficient (β_1_) = 1.038 [coded: ≥3000 yuan = 0, <3000 yuan = 1]
4. Body Image coefficient (β_2_) = 1.353 [coded: High = 0, Low = 1]
5. Social Support coefficient (β_3_) = 0.950 [coded: High = 0, Low = 1]

**FIGURE 1:**
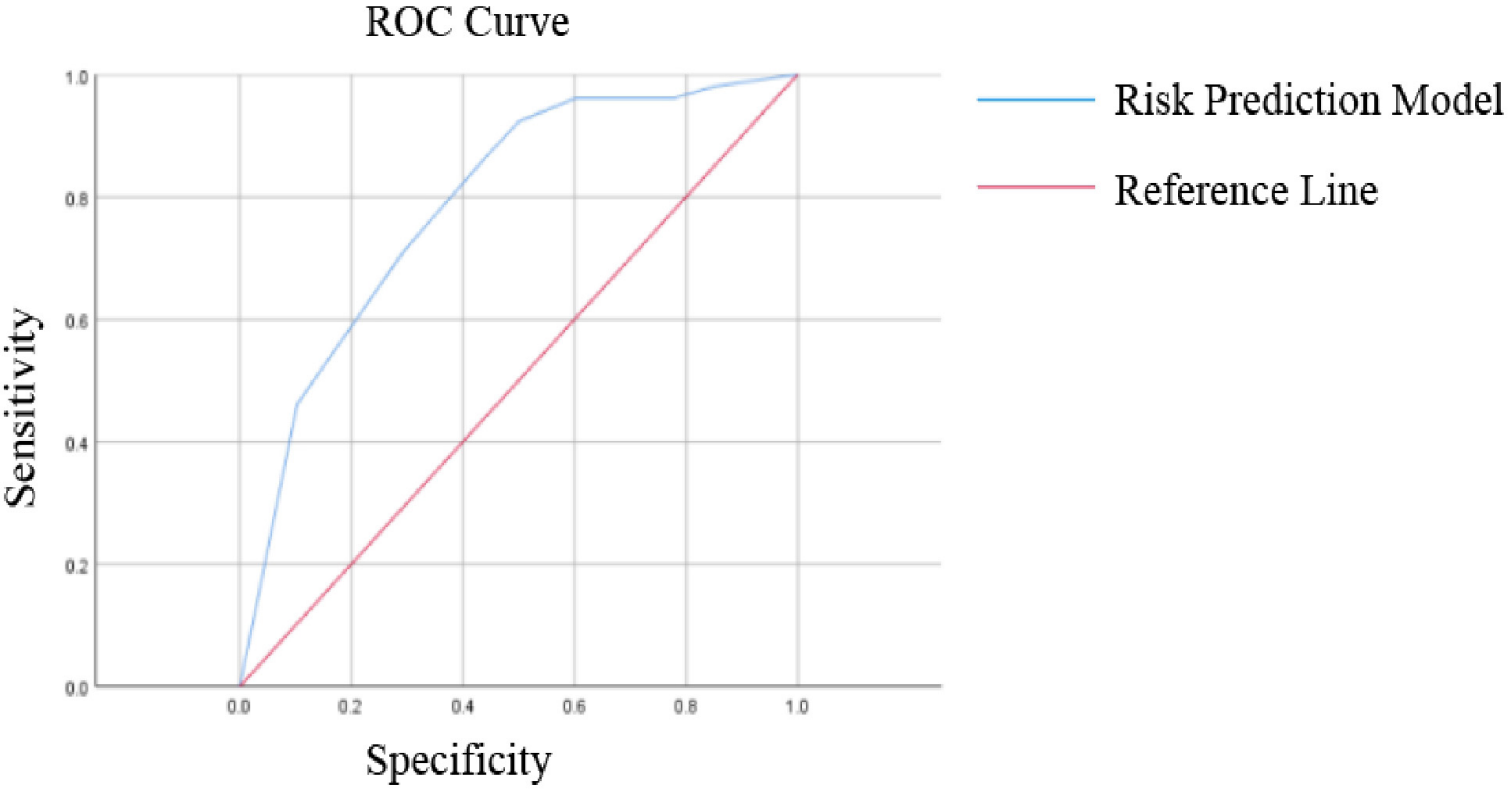
The ROC curve of occurrence of depression in breast cancer patients with postoperative lymphedema.

The probability of cutoff value for classification was 0.155 (15.5%), corresponding to a logit threshold of −1.698. Patients with predicted probabilities ≥ 0.155 were classified as at-risk for depression. The results of the ROC curve analysis showed that the sensitivity and specificity of the model for predicting depression in breast cancer patients with postoperative lymphedema were 86.54% and 55.89%, respectively, with an AUC value of 0.778 (95% CI: 0.707–0.869). (Figure 1 and Tables 3, 4). In the development cohort, the model demonstrated acceptable calibration with a calibration intercept of 0.245 (95% CI: −0.312 to 0.802) and calibration slope of 0.892 (95% CI: 0.534–1.250). The Brier score was 0.213 (Table 5).

**TABLE 3:** Construction of a risk prediction model for predicting depression in patients with lymphedema after breast cancer surgery using ROC curve.

Factors	AUC	95% CI	*P*	Probability cutoff value	Sensitivity	Specificity
Risk assessment model	0.778	0.707∼0.869	<0.001	0.155	86.54 (45/52)	55.89 (38/68)

**TABLE 4:** Variable assignments for multivariate logistic regression analysis.

Variable	Coding rule	Coefficient (β)	
Intercept	–	−3.843	
Family monthly income	≥3000 yuan = 0; <3000 yuan = 1	1.038	
Body image	High body image = 0; Low body image = 1	1.353	
Social support	High social support = 0; Low social support = 1	0.950	
**Non-retained variables**	**Coding rule**	**Coefficient (β)**	***P*-value**
Living arrangement		0.407	0.511
Surgical side	Dominant side = 0; Non-dominant side = 1	0.514	0.272
Lymphedema severity	Mild edema = 0; Severe edema = 1	0.561	0.262
Pain level	None to mild = 0; Moderate to severe = 1	0.276	0.585
Sleep disturbance	Absent = 0; Present = 1	0.363	0.449
Lymphedema knowledge	Good mastery = 0; Poor mastery = 1	0.434	0.419

The following variables were included in initial multivariate analysis but were not retained in the final predictive model due to non-significance (*P* > 0.05).

**TABLE 5:** Calibration metrics for depression risk prediction model.

Metric	Development cohort (*n* = 120)	Internal validation cohort (*n* = 60)
Calibration intercept (95% CI)	0.245 (−0.312 to 0.802)	0.418 (−0.456 to 1.292)
Calibration slope (95% CI)	0.892 (0.534–1.250)	0.756 (0.312–1.200)
Brier score	0.213	0.228
Brier score (null model)	0.247	0.252
Brier skill score	0.138	0.095

### 3.5 Validation results in the internal validation cohort

To assess potential overfitting and preliminary generalizability, the predictive model was tested in the internal validation cohort (*n* = 60) comprising the subsequent 60 consecutive patients enrolled after the development cohort. Among the 60 breast cancer patients with postoperative lymphedema included in the validation cohort, 23 had depression and 37 did not. Using the prespecified optimal cutoff value of −1.698 (probability = 0.155) derived from the development cohort, the risk assessment model achieved an AUC of 0.629 (95% CI: 0.487–0.772) in the validation cohort. The sensitivity and specificity were 82.61% (19/23) and 43.24% (16/37), respectively. Calibration performance remained acceptable in the validation cohort (Table 5). The confusion matrix classification was as follows: 19 true positives, 4 false negatives, 16 true negatives, and 21 false positives (Table 6). In the temporal consecutive validation cohort (*n* = 60), calibration performance was maintained with a calibration intercept of 0.418 (95% CI: −0.456 to 1.292) and calibration slope of 0.756 (95% CI: 0.312–1.200). The Brier score was 0.228, comparable to the development cohort. The calibration plot demonstrated reasonable agreement despite slight attenuation in the slope.

**TABLE 6:** External validation results.

Risk prediction model type	Assessment results		Total	Sensitivity	Specificity
	Depressed	Non-depressed			
Depressed	19 (TP)	21 (FP)	40	82.61	43.24%
Non-depressed	4 (FN)	16 (TN)	20		
Total	23	37	60		

## 4 Discussion

Importantly, this study employed a prospective assessment design where predictor variables were measured at baseline (lymphedema diagnosis) and depression outcomes were evaluated 3 months later. This temporal sequence supports the model’s utility as an early warning tool–identifying at-risk patients during the critical initial adaptation period following lymphedema onset, rather than merely classifying concurrent associations. Breast cancer patients with postoperative lymphedema typically experience symptoms such as limb swelling and pain. The chronic and progressive nature of the condition makes treatment challenging. As a result, patients have to endure physical discomfort over the long term, which can further lead to negative psychological issues like depression. A systematic review by Carreira et al. (18) found that breast cancer patients with postoperative lymphedema who develop adverse psychological conditions such as depression generally have a poorer prognosis. Therefore, it is of great practical significance to focus on the incidence of depression in these patients and to identify independent risk factors in order to develop targeted depression prevention programs.

The results of this study showed that among the 120 breast cancer patients with postoperative lymphedema, 52 had depression, with an incidence rate of 43.33%. This rate is slightly higher than the 29.70% reported by Mete et al. (7) in their study, which may be due to the fact that their study was cross-sectional, and the subjects included may not have been representative enough. However, it is consistent with the 43.40% reported by Hajj et al. (8). These studies also pointed out that younger age, lower cognitive levels, and sleep disturbances are more likely to be associated with depression. The reason for this is that, from a psychobiological perspective, Perez-Tejada et al. (19) found that younger breast cancer patients with depression are more susceptible to the adverse effects of low peripheral dopamine (DA) and serotonin (5-HT). As for lower cognitive levels and sleep disturbances, which fall into the category of social behavior, they can directly or indirectly affect the psychological state of patients. A study by Zhao et al. (10) confirmed that breast cancer patients with postoperative lymphedema still lack basic knowledge about lymphedema and preventive measures after surgery. Over-worrying about the disease during the long-term treatment process can increase the risk of depression. A review of previous literature shows that the factors influencing depression in breast cancer patients with postoperative lymphedema are multifaceted. Breidenbach et al. (20) believed that the type of surgery and comorbidities are closely related to depression in these patients. A multicenter, cross-sectional study by Peng et al. (21) confirmed that the incidence of anxiety and depression in breast cancer patients with moderate to severe lymphedema is significantly higher than that in patients with no too mild lymphedema. Chen et al. (22) showed that comorbidities and living alone can both increase the risk of depression in breast cancer patients with postoperative lymphedema. In addition, some studies have suggested that surgery on the dominant side can exacerbate the degree of depression in patients (23).

This study collected demographic information, clinical parameters, and indicators related to social and cognitive functions for univariate analysis, which confirmed that most of the above factors can have a certain impact on depression in breast cancer patients with postoperative lymphedema. However, the multivariate analysis using the Logistic regression model found that family monthly income (<3000 yuan), poor body image, and low social support are the independent risk factors for depression in these patients. For clinical implementation, the model requires assessment of three readily obtainable variables: family monthly income (<3000 yuan vs. ≥3000 yuan), body image status (evaluated using the Modified Body Image Scale with median split), and social support level (Social Support Rating Scale score < 20 vs. ≥20). The risk score is calculated using the formula: Logit(P) = −3.843 + 1.038(Income) + 1.353(Body Image) + 0.950(Social Support), where each variable is coded as 0 (lower risk) or 1 (higher risk). The resulting probability is compared against the threshold of 0.155 (15.5%) to identify patients requiring enhanced psychological monitoring. This threshold was selected to maximize sensitivity (86.54%) while maintaining acceptable specificity (55.89%) for screening purposes. The specific analysis is as follows: ① Family income: Affected by symptoms such as limited upper limb function and pain, most breast cancer patients with postoperative lymphedema find it difficult to return to work. Among the few patients who do have jobs, long-term treatment can cause delays in resuming work, thereby reducing family income. Facing high medical expenses can lead to negative emotions such as depression (24); ② Body image: When breast cancer patients undergo surgical removal of the breast, there is already a physical defect. The occurrence of lymphedema can further deteriorate body image, making patients more prone to feelings of inferiority and depression in daily life (25). A study by Zhang et al. (26) can corroborate the accuracy of this conclusion; ③ Social support: Due to impaired social functioning in breast cancer patients with postoperative lymphedema, manifested in aspects such as sexual function, interpersonal relationships, and social confidence, these aspects gradually decline over time. In addition, inadequate support from family members can further induce negative emotions such as depression (27). In order to identify depression in breast cancer patients with postoperative lymphedema as early as possible, this study also constructed a risk early warning model. The ROC results showed that the model has good efficacy in predicting depression in these patients, with sensitivity and specificity of 86.54% and 55.89%, respectively. Internal validation in the separate cohort (*n* = 60) confirmed that the model has strong applicability within this institutional population. The similarity in sensitivity (86.54%) between the development and validation cohorts suggests minimal overfitting. However, we acknowledge this represents single-center internal validation only. Temporal consecutive split-sample internal validation suggested preliminary model stability; however, this does not confirm predictive accuracy in diverse clinical settings. Beyond discrimination (AUC), model calibration is essential for clinical decision-making as it ensures that predicted probabilities reliably reflect true event likelihood. Our model demonstrated acceptable calibration in both the development and validation cohorts, with intercepts close to 0 and slopes approaching 1. The slight attenuation of the calibration slope in the validation cohort (0.756 vs. 0.892) suggests mild overfitting, consistent with the modest decline in AUC. The Brier scores of 0.213 and 0.228 indicate good probabilistic accuracy, substantially better than the null model (no predictors). These calibration metrics support the model’s utility for risk stratification, though the modest sample size suggests cautious interpretation at the individual patient level. Future external validation should specifically assess whether predicted probabilities require recalibration in different settings. Our findings align with emerging evidence supporting the clinical utility of predictive risk models in oncology settings (5). While previous studies have established multivariable approaches to depression risk stratification in general cancer populations, our model specifically addresses the unique psychosocial and physical burden of postoperative lymphedema. This specificity enhances clinical applicability for breast cancer survivorship care, where lymphedema represents a distinct risk stratification category requiring tailored psychological monitoring. Prospective external validation across multiple centers is essential before implementation as a clinical screening tool. This suggests that the model can be used to predict the risk of depression in breast cancer patients with postoperative lymphedema. While our model focused on socioeconomic and psychosocial predictors, it is important to acknowledge that breast cancer patients often present with metabolic comorbidities and systemic health burdens that may interact with psychological outcomes (28). Metabolic dysfunction, endocrine alterations, and inflammatory processes associated with breast cancer treatment may represent additional biological pathways influencing depression risk that were not captured in our behavioral assessment framework. Future predictive models should consider integrating biomarkers and metabolic indicators alongside psychosocial factors to enhance predictive accuracy. However, this study has the following limitations: (1) the number of subjects included is relatively small, only 120 cases, which may introduce some bias into the conclusions; (2) since some of the factors influencing depression are related to social behavior and cognition, the quantification based on scale scores in this study may be subjective and could also lead to result bias; (3) our validation was performed using temporal consecutive split-sample allocation (the subsequent 60 patients enrolled after the development cohort), not true external validation from independent centers. While this approach tests for overfitting better than random split or bootstrapping alone, it cannot assess generalizability to different healthcare settings or geographic regions. While this approach tests for overfitting better than random split or bootstrapping alone, it cannot assess generalizability to different healthcare settings or geographic regions. While this approach tests for overfitting better than bootstrapping alone, it cannot assess generalizability to different healthcare settings or geographic regions. Future multi-center external validation studies are needed to confirm the model’s performance in diverse populations. While we established temporality by assessing predictors at baseline and depression outcomes at 3 months, longer follow-up periods (6 or 12 months) would strengthen the prediction of chronic depression trajectories. The current 3-months window represents early detection during the acute adaptation phase. (4) The multivariate model included 9 predictors with only 52 outcome events (EPV ≈ 5.8), which may result in overfitting and overestimated effect sizes. While we employed bootstrapping and split-sample validation to mitigate this, the coefficient estimates may be unstable. (5) The relatively modest sample size (120 development, 60 validation) limited precise estimation of calibration metrics, particularly in the validation cohort where confidence intervals for the calibration slope were wide (0.312–1.200). Future studies should validate this model in larger cohorts or use penalized regression (e.g., LASSO) for variable selection with low EPV ratios. Finally, the observational design and internal validation approach limit causal inference. While our model identifies factors associated with depression risk, we cannot conclude that modifying income status, body image, or social support will prevent depression. “Randomized controlled trials are needed to evaluate whether interventions targeting these modifiable factors reduce depression incidence in this population.”

Treatment-related variables: we acknowledge that adjuvant therapies (chemotherapy, radiotherapy, endocrine therapy) may influence depression risk. However, these treatments were largely completed prior to lymphedema onset in our cohort, and their acute effects on mood would have diminished by the 3-months assessment point. Our analysis focused on modifiable, lymphedema-specific factors that could inform targeted interventions. Future studies should consider including treatment history as a covariate to further clarify its independent contribution.

## 5 Conclusion

This study identified low family income, negative body image, and low social support as factors independently associated with depression in breast cancer patients with postoperative lymphedema. While these findings suggest that psychosocial screening and support interventions may be beneficial, the observational design precludes conclusions regarding causality or preventive efficacy. “The risk assessment model may serve as a screening tool to identify patients requiring closer psychological monitoring; however, external validation in independent cohorts and prospective interventional studies are needed to determine whether modifying these factors reduces depression incidence.”

## Data Availability

The raw data supporting the conclusions of this article will be made available by the authors, without undue reservation.
